# Pathology Survey on a Captive-Bred Colony of the Mexican Goodeid, Nearly Extinct in the Wild, *Zoogoneticus tequila* (Webb & Miller 1998)

**DOI:** 10.1155/2013/401468

**Published:** 2013-10-30

**Authors:** Alessio Arbuatti, Leonardo Della Salda, Mariarita Romanucci

**Affiliations:** Faculty of Veterinary Medicine, University of Teramo, Piazza Aldo Moro 45, 64100 Teramo, Italy

## Abstract

The Mexican Goodeid, *Zoogoneticus tequila*, is considered nearly extinct in the wild and it is maintained in captivity by the nonprofit international “Goodeid Working Group.” The unique Italian colony has produced about 180 fish so far. The observable diseases were registered and some fish were submitted, immediately after spontaneous death, to necroscopic and histopathologic exams. Encountered diseases included the following: 7 cases of scoliosis (2 males and 5 females); 2 fish with a similar congenital deviation of ocular axis; 1 adult male with left corneal opacity, presumably of traumatic origin; 1 female fish with a large subocular fluid-filled sac, histologically referable to a lymphatic cyst, similarly to the eye sacs of a Goldfish variety (*Carassius auratus*) called *bubble eye*; and 1 female fish with recurrent abdominal distension consequent to distal bowel dilation and thinning, associated with complete mucosal atrophy, and comparable to intestinal pseudo-obstruction syndromes described in humans and various animal species. The absence of infectious or parasitic diseases, as well as the low incidence of diseases potentially related to environmental alterations or nutritional disorders such as spinal deformities, suggests the adequacy of breeding management techniques of *Z. tequila* for its conservation and reintroduction in to the original habitat in the near future.

## 1. Introduction 

**Figure 1:**
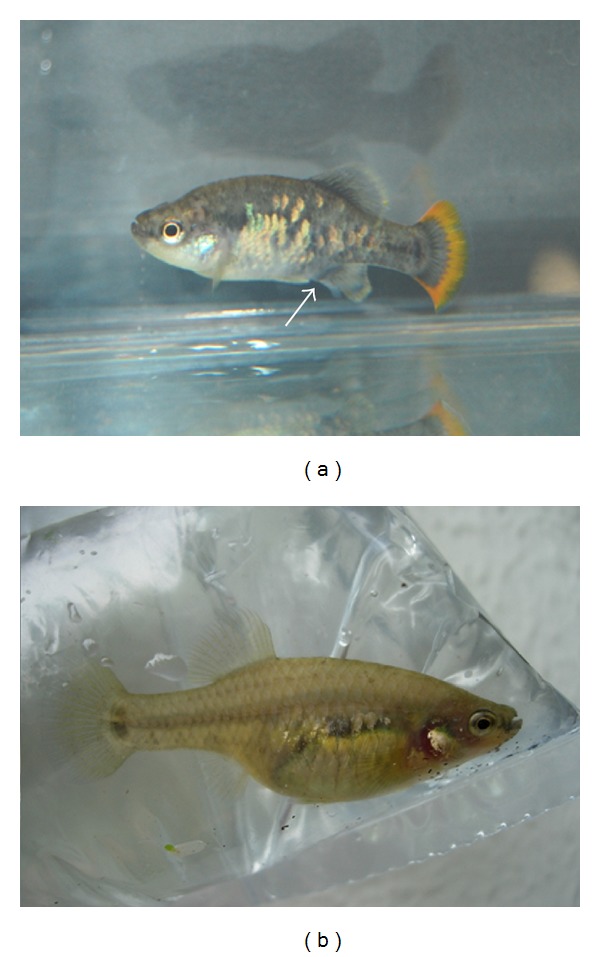
Adult male *Z. tequila* showing the typical orange-yellow band in the caudal fin and the andropodium (arrow), a copulatory organ allowing the transfer of spermatozeugmata (sperm bundles) in the female genital apparatus (a); adult female *Z. tequila*. Females give birth to free-swimming fry after an intraovarian gestation (b).

The Goodeidae family (Cyprinodontidae) is a characteristic component of the ichthyofauna of Central Mexico, consisting of around 36 species of viviparous topminnows, which are sexually dimorphic and largely omnivorous. Unfortunately, due to a combination of habitat loss from anthropogenic impacts, introduction of exotic fish, and their being a species with restricted physiologic tolerance, Goodeid fish have often been reported as extinct or extirpated from the wild [1]. In particular, *Zoogoneticus tequila* (Figure 1) is native to the Teuchitlan River in the Ameca basin, which has experienced massive changes following the building of a dam in 1955. This species was thought to be already extinct in the wild when it was first formally described from captive populations and museum specimens in 1998 [2]. Many scientists and aquarists also continue to believe that no wild populations exist [3]. However, in 2000-2001, a remnant wild population was discovered within a very small and isolated pond at the Teuchitlan springs. This population was composed of only a handful of adult fish and a few tens of juveniles [4], which also exhibited a poor genetic variability [5], thus representing an extreme case of vulnerability to extinction. It has been suggested that habitat degradation may be the reason why the extant endemic Goodeids are now confined to a small area at the headwaters of the river. The small pool in which this species was rediscovered is also threatened by pollution and water extraction. Therefore, due to its limited extent of occurrence and restricted area of occupancy, *Z. tequila* is classified as critically endangered (IUCN 2009). This species is being maintained in captivity by zoological institutions and aquarists in North America, Mexico, and Europe, which are organized into a nonprofit international working group called the Goodeid Working Group. It has been recommended that, in addition to efforts to recover a substantial portion of its original environment [5], breeding programmes should be implemented under the supervision of international conservation agencies so that *Z. tequila* can be reintroduced into the original, larger pools previously inhabited by this species [6]. In this respect, in 2011, the Goodeid Working Group started a reintroduction project for this species. In 2012, the programme built semicaptive ponds for the acclimation of captive-bred fish, allowing their reintroduction and scientific monitoring over 2 years. The biology of *Z. tequila* in the wild is unknown, and information concerning its optimal management in captivity is also limited [7]. In addition, no literature reports regarding pathologies eventually encountered in captive populations of this species are available. 

Aquarist experiences in keeping Goodeids may provide scientists and conservationists with essential information on Goodeid biology and pathology that cannot be determined from remaining wild populations and may be useful for their proper reintroduction into the original habitat. 

## 2. Materials and Methods 

The unique, currently existing Italian colony was founded in 2007 by one of the authors (AA, regional group coordinator for Italy within the Goodeid Working Group) from 14 specimens obtained from Amboise France. Fish were bred in different structures including two tropical freshwater planted aquariums (180 L and 250 L) housing the main colony, an outdoor pond (360 L) used for breeding and reproduction during the warm season (from June to September), and a multitank aquarium system (180 L) used for fry and young fish growth [7]. Routine management provided a weekly water change (20%), using properly bioconditioned water, enriched with filter bacteria, and a daily administration of high quality dry (SHG Microgranules, SHG Spirulina, SHG *Artemia salina* flakes, Sera FD Tubifex, Sera Daphnia, and Sera Artemia shrimps), frozen (*Artemia salina*, *Tubifex* sp., and mosquito larvae), and live (*Enchytraeus buchholzi*) foods. Pathologies observed during breeding management were recorded and some fish showing macroscopically evident lesions, as well as some control fish, were submitted for necropsy immediately after spontaneous death that occurred at advanced age. Samples of tissues with gross lesions, as well as samples of macroscopically normal organs including gills, heart, liver, kidneys, and intestine, were fixed in 10% neutral buffered formalin, embedded in paraffin wax, and sectioned at a thickness of 5 *μ*m. Deparaffinized sections were finally stained with haematoxylin and eosin (H&E).

## 3. Results 

**Figure 2:**
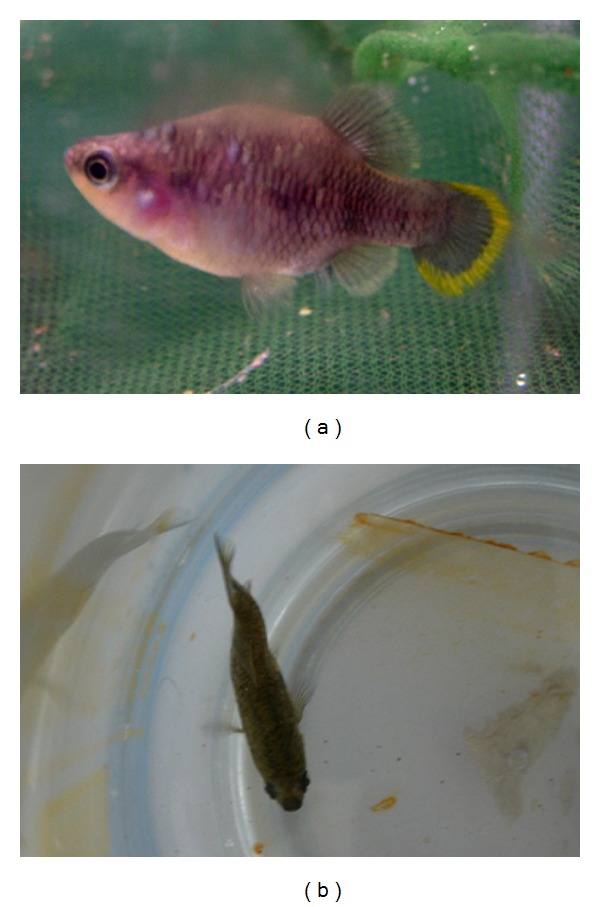
Lateral (a) and dorsal (b) views of an adult male showing scoliosis.

**Figure 3:**
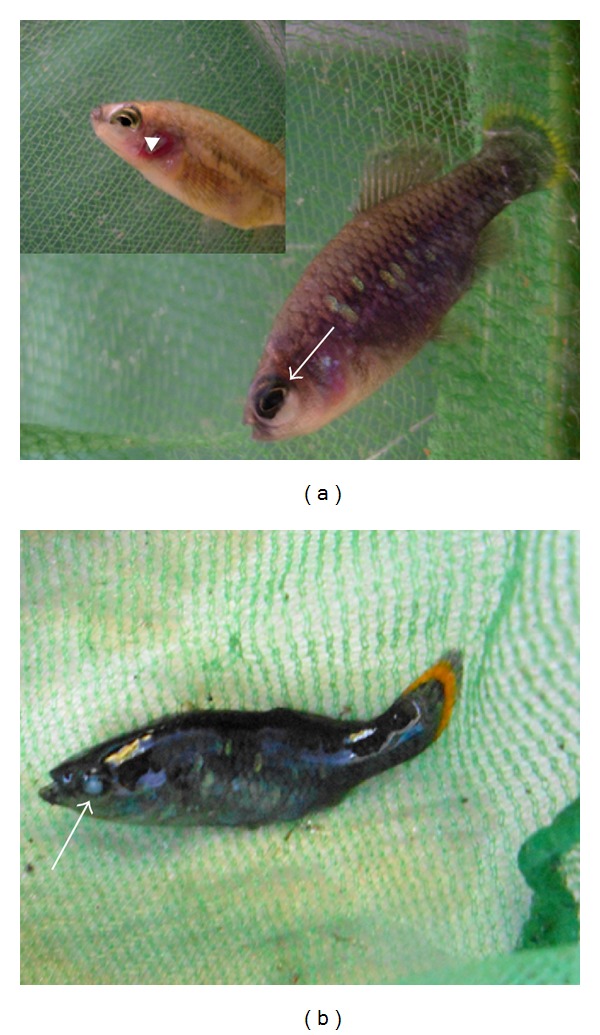
Adult male (arrow) and female (inset, arrowhead) fish with a similar congenital deviation of left ocular axis (a); adult male with left corneal opacity (arrow) and diffuse dark colour (b).

**Figure 4:**
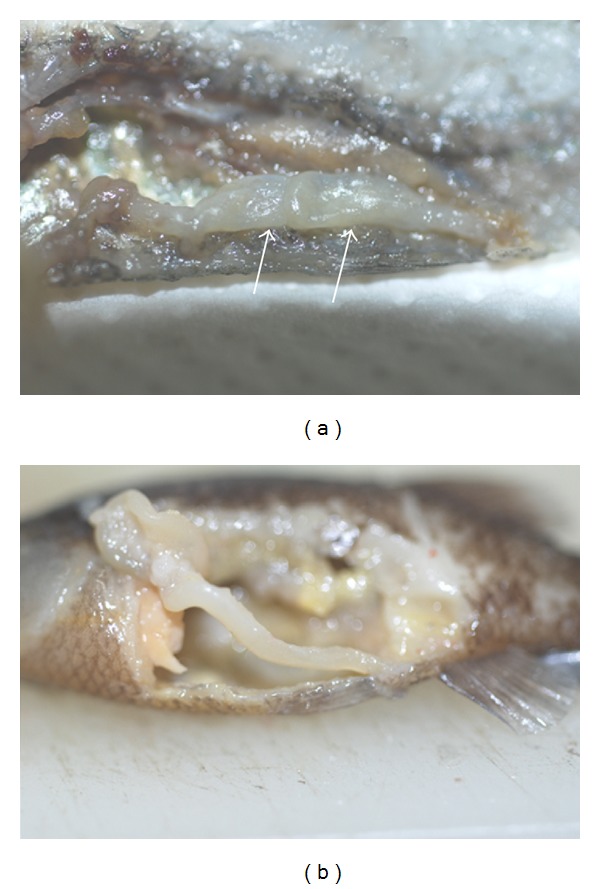
Dilated distal intestine (arrows) of an adult female with recurrent abdominal distension (a); normal distal intestine of a control fish (b).

**Figure 5:**
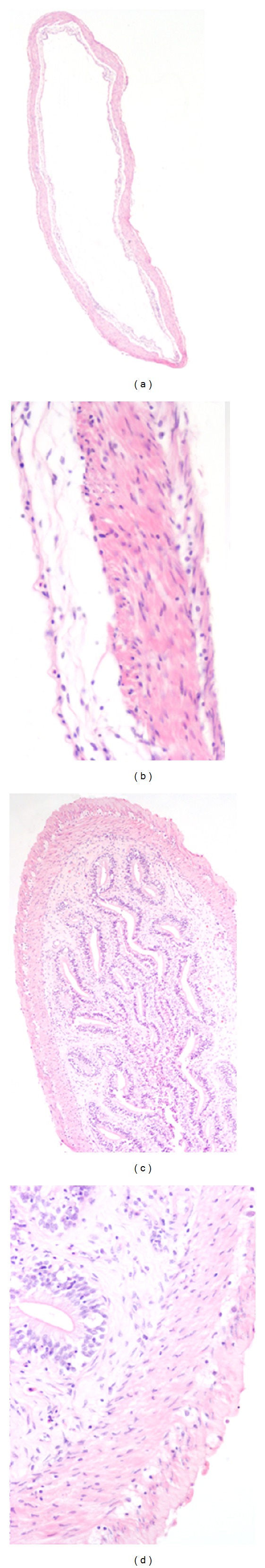
*Z. tequila* with recurrent abdominal distention: dilated intestinal tract showing complete mucosal and submucosal atrophy with fold disappearance. Moderate thinning of the outer longitudinal layer of the tunica muscularis is also observable ((a)-(b)); adjacent normal intestinal tract ((c)-(d)) (H&E, (a)–(c) 10x, (b)–(d) 40x).

Out of a total of about 180 fish, the following pathologies were recorded: 7 cases of fish with spinal deformities (scoliosis in 2 males and 5 females), all of them showing normal growth, swimming, and breeding behaviour (Figure 2); 2 cases of fish (1 male and 1 female) with a similar congenital eye defect, characterized by an internal deviation of the ventral part of left ocular axis (Figure 3(a)); 1 case of an adult male with left corneal opacity, presumably resulting from posttraumatic keratitis, which also darkened in colour, probably as a consequence of partial blindness (Figure 3(b)); 1 case of a female fish with recurrent abdominal distention. At postmortem examination, a segmental, marked dilation and thinning of the distal intestine was observed (Figure 4(a)). Intestines of control fish appeared as simple tubes with a uniform diameter (Figure 4(b)). Histologically, the dilated tract showed complete mucosal and submucosal atrophy, characterized by the presence of a thin layer of loose connective tissue with the disappearance of intestinal epithelium, as well as a moderate thinning of the outer longitudinal layer of tunica muscularis (Figures 5(a)-5(b)). Loss of intestinal epithelium due to autolysis was excluded, since the other parts of digestive tract, grossly appearing normal, showed a complete intestinal wall, provided with long intestinal folds (Figures 5(c)-5(d)), as it was observable in the intestine of control fish. 

**Figure 6:**
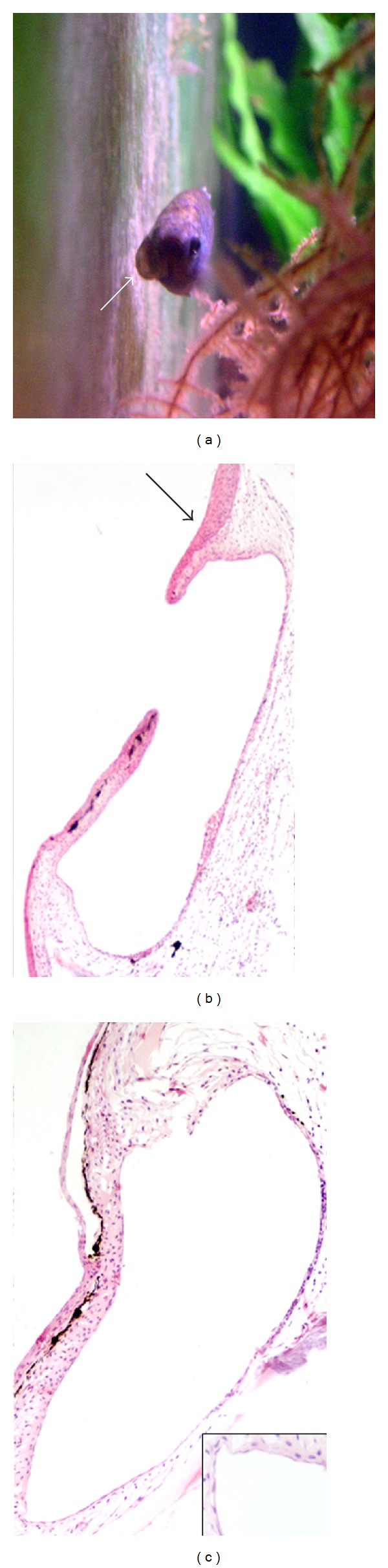
Adult female with a sub-ocular fluid-filled sac (arrow) (a); cystic cavity of the sac in continuity with the overlying hyperplastic epidermis (arrow) at the rupture site of the cyst wall (b); dermal lymphatic vessel located at the opposite cranium side; *inset*: magnified view of the endothelial lining of the lymphatic vessel (c) (H&E, (b) 10x, (c) 20x, *inset* 40x).

Another case concerned a female fish with a large subocular fluid-filled sac below the right eye (Figure 6(a)), associated with progressive cataract formation in the ipsilateral eye. This sac accidentally ruptured, with disappearance of dilation, but without further consequences for the fish, except for a slight cranial asymmetry. Histological examination revealed a large dermal cavity lined by flattened endothelial-like cells, in places obscured by the presence of a moderate lymphocyte infiltration in the cystic wall and surrounding dermis, and in continuity with the overlying hyperplastic epidermis at the rupture site of the cystic wall (Figure 6(b)). On the opposite side of the cranium, a large vascular space lined by endothelial cells was observed that was identified as a lymphatic vessel surrounded by loose dermal tissue (Figure 6(c)). 

Clinical evaluation of fish performed during routine management of aquariums, as well as necroscopical and histopathological examinations, did not reveal any occurrence of infectious or parasitic diseases. 

## 4. Discussion 

Spinal deformities are commonly observed in both wild and farmed fish, due to a multifactorial etiology, including a variety of injuries which can be classified as physical, environmental, nutritional, infectious, and genetic/heritable [8, 9]. Even though the exact cause of spinal curvatures observed in the present survey was not determined, it is important to notice that their incidence was very low (about 3.8%), thus providing useful information on the optimal maintenance of *Z. tequila* in captivity, since increased incidence of skeletal deformities may be a signal of environmental deterioration that indicates the need for timely corrective actions [10]. In addition, the limited occurrence of congenital defects also was a favorable finding, since congenital anomalies are particularly frequent in inbred populations [11], and allelic diversity of captive stocks of *Z. tequila* has been found to be lower than that of wild population [5]. 

The case of recurrent abdominal distension due to segmental dilation of distal intestine appears to be comparable to intestinal pseudo-obstruction syndromes, rarely described in humans and various animal species [12–14]. However, no literature reports are available for fish species. In human medicine, chronic intestinal pseudo-obstruction is a rare but well-known disease, which can be focussed in one part of the intestine or affect larger regions, and is characterized by chronic or recurrent symptoms of intestinal obstruction without any obvious mechanical cause [15]. Although the majority of cases appear to be idiopathic (primary), neuropathic or myopathic disorders may be involved in the pathogenesis of this condition [12, 14]. Observable histological lesions were also variable, being absent in some cases, or characterized by inflammatory or degenerative alterations of the myenteric plexus or by atrophy and degenerative lesions of smooth muscle cells of the tunica muscularis [14, 16–19]. In our cases, we did not observe inflammatory infiltrations in the dilated intestinal wall. Histopathological evaluation of the tunica muscularis did not reveal the existence of muscular abnormalities except for a moderate thinning of the outer longitudinal layer. Immunohistochemistry might have been useful to assess the integrity of enteric nervous system and smooth muscle [12, 15], but it was inapplicable in our case, since available antibodies directed against neuronal or smooth muscle markers did not cross-react with fish tissues (data not shown). Notwithstanding this, a severe villous atrophy is a well-known event in these syndromes, which could be due to intraluminal factors, such as bacterial overgrowth and toxic bacterial metabolites [20, 21]. 

In the case of a female fish with a subocular fluid-filled sac, histological examination revealed a lymphatic origin (lymphatic cyst), comparable to that of the fluid-filled eye sacs typical of a Goldfish variety (*Carassius auratus*), called *bubble eye*, which are known to contain lymph, which is similar in composition to serum or blood plasma [22]. The thin wall of these eye sacs may easily rupture, with consequent bacterial infection; however, this event did not occur in our case. 

## 5. Conclusions 

This pathology survey revealed the absence of infectious or parasitic diseases. There also was a low incidence of diseases potentially related to husbandry procedures, environmental alterations, nutritional imbalances, or inbreeding, such as spinal curvatures or other physical deformities. These findings support the suitability of the breeding management of *Z. tequila* in captivity for the conservation of this species and its possible reintroduction into the natural habitat in the near future.
